# The impact of humanitarian aid on financial toxicity among cancer patients in Northwest Syria

**DOI:** 10.1186/s12913-024-11077-x

**Published:** 2024-05-18

**Authors:** Orwa Al-Abdulla, Aliye Aslı Sonsuz, Maher Alaref, Bakor Albakor, Jussi Kauhanen

**Affiliations:** 1 Institute of Public Health and Clinical Nutrition, Faculty of Health Sciences, The University of Eastern Finland, P.O. Box 1627, Kuopio, 70211 Finland; 2 Strategic Research Center (Öz SRC), Incili Pinar MAH, Gazi Muhtar Paşa BUL, Doktorlar Sitesi, 38E, 104. Sehitkamil, 27090 Gaziantep, Türkiye; 3 Health Science Institute, Istanbul Medipol University, Beykoz, İstanbul Türkiye

**Keywords:** Cancer, Conflict, Financial toxicity, Humanitarian, Impact, Syria

## Abstract

**Introduction:**

The ongoing crisis in Syria has divided the country, leading to significant deterioration of the healthcare infrastructure and leaving millions of people struggling with poor socioeconomic conditions. Consequently, the affordability of healthcare services for the population has been compromised. Cancer patients in Northwest Syria have faced difficulties in accessing healthcare services, which increased their financial distress despite the existence of humanitarian health and aid programs. This study aimed to provide insights into how humanitarian assistance can alleviate the financial burdens associated with cancer treatment in conflict-affected regions.

**Materials and methods:**

This research employed a quantitative, quasi-experimental design with a pre-test-post-test approach, focusing on evaluating the financial toxicity among cancer patients in Northwest Syria before and after receiving humanitarian aid. The study used purposeful sampling to select participants and included comprehensive demographic data collection. The primary tool for measuring financial toxicity was the Comprehensive Score for Financial Toxicity (FACIT-COST) tool, administered in Arabic. Data analysis was conducted using SPSS v25, employing various statistical tests to explore relationships and impacts.

**Results:**

A total of 99 cancer patients were recruited in the first round of data collection, out of whom 28 patients affirmed consistent receipt of humanitarian aid throughout the follow-up period. The results of the study revealed that humanitarian aid has no significant relationship with reducing the financial toxicity experienced by cancer patients in Northwest Syria. Despite the aid efforts, many patients continued to face significant financial distress.

**Conclusion:**

The research findings indicate that current humanitarian assistance models might not sufficiently address the complex financial challenges faced by cancer patients in conflict zones. The research emphasizes the need for a more comprehensive and integrated approach in humanitarian aid programs. The study highlights the importance of addressing the economic burdens associated with cancer care in conflict settings and calls for a re-evaluation of aid delivery models to better serve the needs of chronic disease patients. The findings suggest a need for multi-sectoral collaboration and a systemic approach to improve the overall effectiveness of humanitarian assistance in such contexts.

**Supplementary Information:**

The online version contains supplementary material available at 10.1186/s12913-024-11077-x.

## Introduction

Low and Middle-Income Countries (LMICs) or resource-limited countries are characterized by their constrained resources for advanced health services provision, health system governance, and health planning. Consequently, there has been an observable escalation in both the incidence and mortality rates associated with cancer in these regions. [1] [2] [3]. All health systems face the challenge of addressing health needs amidst limited resources, a situation that is particularly pronounced in low-income countries. These countries are characterized by rapid population growth and markedly limited financial allocations for health, compounded by constrained resources for the provision of advanced health services, as well as challenges in health system governance and planning [4] [5].

Cancer patients in these regions often confront a myriad of hurdles when seeking care and treatment. These challenges range from insufficient social support, financial pressures, and a lack of comprehensive medical insurance to communication obstacles [6]. Furthermore, the health insurance frameworks in numerous LMICs either remain underdeveloped or are not broadly applicable, leaving many patients unprotected against the rising costs of cancer treatment [7].

The socioeconomic burden of cancer treatment is significant and results in catastrophic health expenditure (where treatment costs exceed 40% of a household’s capacity to pay) and financial distress, characterized by a subjective perception of economic well-being leading to a lower sense of control over life) [8] [9] [10]. Introduced in 2009, the term “financial toxicity” was used to elucidate the economic repercussions of cancer treatment on patients [11]. This concept, which become integral to discussions on cancer care, can manifest in various ways, from the subjective distress arising from routine copayments unsettling household finances to the extreme scenario of personal bankruptcy [12] [13]. Several instruments, such as the Breast Cancer Finance Survey Inventory, the Socioeconomic Well-being Scale, and the COmprehensive Score for financial Toxicity (COST)-Functional Assessment of Chronic Illness Therapy (FACIT) (COST-FACIT, or COST questionnaire), have been employed to gauge this financial distress [14].

Syria, a low-income country currently [15], has been experiencing an armed conflict between the government and opposition groups since 2011 [16]. The protracted war in Syria devastated the health system and tore the country into many regions controlled by the government and armed groups [17] [18]. A humanitarian coordination mechanism was established through humanitarian organizations and United Nations agencies to address the needs of affected people in areas outside Syrian government control in Northwest Syria (NWS) [19]. NWS refers to the territories in Idleb and Aleppo governorates that are under the control of opposition armed groups. These territories are home to a population of over 4.5 million, with more than half being Internally Displaced Persons (IDPs) [20] [21]. This geographical region of Syria is characterized by a fragmented health system and administrative division, setting it apart from the rest of Syria [22]. Its unique geographical location is bordered by Türkiye and isolated from other Syrian territories controlled by the government. This isolation necessitates reliance on cross-border initiatives from Türkiye for the delivery of healthcare and other humanitarian aid [23]. Nowadays, the health system and service delivery provision in NWS are sustained mainly by humanitarian financial support sourced from multiple donors through various funding mechanisms [24].

Similar to other LMICs affected by humanitarian crises, there is a scarcity of information regarding the burden of cancer care and treatment in Syria and limited guidance on addressing its impact [25]. In fact, we could not find any study addressing financial toxicity in Syria. Additionally, the literature lacks publications regarding the impact of humanitarian assistance on financial toxicity during emergencies and how multi-sectoral humanitarian aid contributes to improving the socioeconomic and public health conditions among cancer patients. The research on the effects of conflict or crisis on cancer incidence and mortality is limited, methodologically deficient, and frequently contradictory. There is an urgent need to address this “data poverty” and initiate more rigorous longitudinal and cohort studies of conflict-affected populations to guide the development of basic cancer care recommendations and post-conflict cancer control planning [26].

Recent data pertaining to cancer patients in NWS may indicate a more critical situation relative to regional benchmarks. A predominant portion of these patients is unable to access sufficient treatment within the region due to the lack of specialized medical workers and required medical devices, necessitating referrals to medical facilities in southern Türkiye [27]. The earthquake that struck southern Türkiye and NWS in February 2023 highlighted the fragility of cancer patients, particularly children, due to disruptions in the provision of medical care in NWS and referral services to Türkiye [28]. The interruption of medical referrals from NWS to Türkiye because of the devastating earthquake critically impeded cancer patients’ access to essential care services available in Türkiye. This situation increased the urgency of strengthening local healthcare services to ensure the continuity of cancer treatment within the region of NWS [29]. Moreover, while humanitarian efforts may cover certain direct costs associated with cancer treatment, there is a gap in addressing the financial burden due to cancer care. Expenses related to travel and accommodation for referred patients in Türkiye, interrupted referral services, and poor socioeconomic conditions emerge as substantial burdens. Additionally, the lack of diagnostics and treatment services in NWS further exacerbates the economic strain on patients. These challenges illuminate the complex nature of financial toxicity and the need for comprehensive aid strategies that extend beyond the coverage of medication costs to encompass the broader economic challenges faced by cancer patients in conflict-affected areas [30] [31]. A cross-sectional study from Syria in 2016 found that the expensive cost of cancer treatment might prohibit cancer patients from attaining the required treatment and care. The study showed that the monthly costs of cancer treatment range from 100$ to 1000$, which is relatively expensive considering the dire socioeconomic conditions in Syria due to the crisis. The high costs of cancer treatment, besides the limited resources, affect the quality of care by providing limited amounts and cheaper or alternative pharmaceuticals [27].

In addressing the crucial aspect of financial toxicity within the local context of NWS, this study emphasizes the importance of having a detailed understanding to fully capture and address the complex nature of financial burdens faced by cancer patients in a region affected by conflict. Recognizing the limitations of existing tools in fully encapsulating the local socioeconomic realities [14], we utilized COST-FACIT tool. We adapted this tool by including context-specific considerations such as the impact of displacement, the availability of humanitarian aid, and the indirect costs associated with seeking cancer care in a conflict zone. These adaptations aim to bridge the gap between the universal application of financial toxicity measurements and the unique experiences of cancer patients in a humanitarian context, like NWS. By incorporating local insights and considering the broader socioeconomic impact of cancer within this setting, our approach seeks to enhance the relevance and applicability of our findings, providing a more comprehensive understanding of financial toxicity deeply rooted in the local context of NWS. This distinct perspective not only enriches the academic discourse on financial toxicity but also serves as a solid foundation for the development of targeted interventions and policies designed to alleviate the economic hardships faced by cancer patients in conflict-affected regions.

Despite the presumption of available humanitarian aid for cancer patients in NWS, it is posited that such assistance may not substantially mitigate the financial burden and subjective distress associated with their medical conditions. Consequently, the hypothesis advanced is that humanitarian aid exerts no significant impact on the financial toxicity experienced by cancer patients in NWS. This research aims to explore financial toxicity in NWS, a conflict-affected region, measure the impact of humanitarian aid on financial toxicity among cancer patients, and investigate potential factors affecting financial toxicity. In fact, this research is the first to address this topic, not only in Syria but also globally.

## Materials and methods

### Study design, setting, and participants

We performed a quantitative method study with a purposeful sampling design to recruit cancer patients for the data collection. This research was planned with a single-group pre-test-post-test design, one of the quasi-experimental research designs. Such studies are used to estimate the causal effect of the intervention on the target population without random assignment. In this design, the experimental group is measured (pre-test) before the intervention, then the intervention is performed, and after the intervention, the same group is measured again (post-test) to determine the difference[32]. At the time of writing this research, humanitarian aid and health care, including cancer treatment, in NWS had been delivered through humanitarian organizations and actors [33]. According to the health cluster for the humanitarian response in NWS, there are three diagnosis and treatment oncology centers operated by one humanitarian organization that deliver free-of-charge health services (Al Abdulla O, personal communication, August 12, 2022). This study investigates the impact of multiple modalities of humanitarian assistance on the financial toxicity of cancer patients registered and receiving cancer treatment in these health facilities. Humanitarian assistance is defined as “the active provision of aid designed to save lives, alleviate suffering, and restore and promote human dignity in the wake of disasters and during large-scale emergencies.” [34].

The sample inclusion criteria were (1) age of at least 18 years, (2) a confirmed diagnosis of any malignant tumor of any stage at the existing Oncology centers in NWS, (3) participation in therapy for at least two months at the time of the data collection, (4) the patient has not received certain types humanitarian assistance for the past two months at least, and (5) the patient consent to be recruited for data collection. Patients with language difficulties, cognitive impairment, and communication challenges were excluded.

### Study variables

Comprehensive demographic data about age, race, sex, profession, marital status, level of education, average monthly income in USD, employment status, dwelling status (camps and informal residence dwelling, and formal residence dwelling), and being an IDP or not were collected. Independent variables are grouped under two main headings. The first one belongs to the sociodemographic characteristics of the participants, and the second is the types of humanitarian aid they receive. The COST value, the dependent variable, was estimated using the COST tool. This tool was translated into Arabic and administered face-to-face by trained staff. The data collection team was composed of qualified males and females to ensure sufficient interaction among female patients. To measure the impact of humanitarian assistance and how the delivery of aid in a humanitarian context could contribute to reducing financial toxicity, we chose the types of humanitarian assistance that contribute directly and indirectly to enhancing household income and living and socioeconomic conditions. Cash-based humanitarian assistance, food distribution, hygiene kits, and non-food items distribution were selected for this study based on available evidence of the relationship between these types of aid and socioeconomic and livelihood status in humanitarian settings [35] [36] [37] [38] [39] [40] [41].

### Data collection and analysis

Data collection was conducted employing two distinct questionnaires. The initial questionnaire was specifically designed for this study, aiming to gather sociodemographic data, details regarding the health status of participants, and information pertaining to the receipt of humanitarian aid by the patients. The first questionnaire is available in the supplementary documents. The subjective distress was measured and interpreted as financial toxicity using the COST-FACIT version 2 questionnaire [42] (available at: https://www.facit.org/measures/FACIT-COST). The COST-FACIT questionnaire was developed by De Souza et al. and was validated to assess the degree of financial stress experienced by patients with cancer [43] [44]. The COST tool consists of 11 items with a 5-point Likert scale from 0 to 4. The COST value (financial toxicity), therefore, ranges between 0 and 44, and a higher score indicates better financial well-being [45] [46]. We used a grading system (financial toxicity grades) that links the level of financial toxicity and the COST value: Grade 0 (No financial toxicity for a COST value ≥ 26), Grade 1 (mild financial toxicity for a COST value between 14 and 25), Grade 2 (moderate financial toxicity for a COST value between 1 and 13), and Grade 3 (severe financial toxicity for a COST value of 0). In this context, an increase in the financial toxicity grade corresponds to experiencing increased subjective distress [43,47].

Data collection was performed in two stages. First, we identified the cases based on the inclusion criteria and measured the financial toxicity. Data were collected in November and December 2022 based on the COST-FACIT tool guidelines [42]. Later, we tracked the patients for eight to ten months. The interval between the two distribution rounds was suggested to allow a sufficient period for humanitarian aid to have a discernible effect on the people’s living conditions. Those who confirmed they regularly received the selected kinds of humanitarian assistance throughout the follow-up period were recruited again for another round of interviews employing the COST-FACIT questionnaire. Data collectors were selected based on experience, gender, and ability to use the Kobo toolbox, a data collection and management platform specific to humanitarian action, development, environmental protection, peacebuilding, and human rights. The data collectors receive training in several key areas, including utilizing the Kobo toolbox, ethical data collection practices, cultural sensitivity and awareness, the consent process, and interview techniques. As part of this research project, they also received training on how to ethically interact with cancer patients and how to appropriately communicate with the medical team in the event of any medical complications that may arise during the interview process. The questionnaire was uploaded to the Kobo toolbox to be administered electronically by the data collectors. The data were synchronized to a database platform administered by the researcher responsible for data analysis and interpretation.

SPSS v25 was used for data analysis to investigate significant relationships and correlations between the demographic data and financial toxicity. Chi-squared test χ^2^, Fisher Exact test, and Sparsman and Pearson correlation tests were applied to measure the statistical relationship and correlation between the variables. Additionally, the Wilcoxon Signed-Rank test was used to measure the impact of humanitarian aid on financial toxicity levels because the relevant data were not normally distributed [48]. All statistical analyses were performed on a Confidence Interval CI level of 95% and a margin of error of 0.05. Questionnaires, anonymized row data, and data analysis documents were uploaded to the Mendeley Data repository https://data.mendeley.com/datasets/dsktsd99g3/1.

### Measuring the impact of humanitarian aid on financial toxicity

The assessment involved applying a grading system to the COST values of each patient across the two phases of data collection. Subsequently, the variance in scores between the rounds was determined by subtracting the initial round’s COST value from that of the second round. Our research has suggested a novel grading scale linked to the COST tool to quantify and measure the impact of humanitarian aid on financial toxicity. This scale categorizes the level of impact into four distinct grades based on the calculated COST value difference value. ‘No Impact’ for a differential score of 0 or less, ‘Mild Impact’ if the difference value was between 1 and 13, ‘Moderate Impact’ if the difference value was between 14 and 25, and ‘Significant Impact’ if the difference value was 26 and more. We called this innovative grading system the Impact Grading System of Humanitarian Aid on Financial Toxicity. This system is an empirical attempt to apply a quantitative measure to assess such impacts in the field of humanitarian aid research.

### Ethical aspects

Written ethical approval for the data collection was obtained from Istanbul Medipol University, Türkiye, and the letter was translated into Arabic and notarized. Additionally, permission to use the questionnaire was obtained from FACIT. A consent form was taken from every patient in the two stages. Written approval was also taken by emails from the humanitarian organization operating the oncology centers in NWS. A specialized doctor was consulted in advance about the patient’s medical condition if they were able and conscious enough to be interviewed. The cases were anonymized before their information was stored in the database. Female data collectors were recruited to collect data from female patients, considering the culture and traditions in the NWS region.

## Results

### COST value: the first phase of data collection

A total of 110 subjects were approached for the interview, of whom 105 consented to participate. From those, 99 met the inclusion criteria and were thus enrolled for data analysis (the sample size *N* = 99). The participant list comprised 58 females and 41 males, and all of them completed the items of The COST-FACIT questionnaire. Participants’ ages ranged from 18 to 83 years, with an average age of 50 and a median of 52. Among these, 46 individuals were identified as IDPs, with 22 residing in camps or informal dwellings. Out of the total participants, 79.8% do not pay for accommodation.

Regarding marital status, 78.8% were married, 9.1% were single, 10.1% were widowed, and 1% were divorced. Literacy levels varied, with 65.7% of participants lacking any formal education, 28.3% having completed secondary education, and 6.1% possessing higher education. Unemployment was prevalent among the participants, standing at 96%. The average household number of individuals was reported as 7 members, with an average monthly income of 87 USD. The employment of at least one member within the household was confirmed by 57.6% of respondents. More than half of the participants, 53.3%, reported receiving financial assistance from family or relatives to manage living expenses and the costs associated with cancer therapy. Among the subjects, 82 indicated that they typically incurred out-of-pocket expenses for cancer treatment. Table 1 below shows the sociodemographic profile of the research sample disaggregated by sex.

**Table 1:** Demographics figures of the first round participants disaggregated by sex

Variables		Participants sex				Total *N* = 99
		Male		Female		
Dwelling status	Not an IDP	19	35.8%	34	64.2%	53
	an IDP	22	47.8%	24	52.2%	46
Marital status	Married	35	44.9%	43	55.1%	78
	Single	5	55.6%	4	44.4%	9
	Divorced	0	0.0%	1	100.0%	1
	Widow	1	10.0%	9	90.0%	10
	No answer	0	0.0%	1	100.0%	1
Level of education	Higher than secondary education	5	83.3%	1	16.7%	6
	Secondary school and lower	13	46.4%	15	53.6%	28
	illiterate	23	35.4%	42	64.6%	65
Employment	No	37	38.9%	58	61.1%	95
	Yes	4	100.0%	0	0.0%	4
Do you pay rent for accommodation	No	31	39.2%	48	60.8%	79
	Yes	10	50.0%	10	50.0%	20
Do you pay for cancer treatment	No	7	41.2%	10	58.8%	17
	Yes	34	41.5%	48	58.5%	82
do your family or relatives financially support you to cover the treatment or life expenses	No	17	58.6%	12	41.4%	29
	Yes	17	32.1%	36	67.9%	53

*N* = 99 (the first round of data collection)

In the initial analysis, the mean COST value of all the patients was 7, reflective of a moderate level of financial toxicity. Further analysis revealed heterogeneity in financial toxicity grades, with 40 individuals experiencing severe financial toxicity, 35 with moderate toxicity, and 20 with mild toxicity. The results showed that only 4 patients were with no financial toxicity. Data analysis revealed a notable association between gender and financial toxicity grades, as evidenced by a *χ*^*2*^ value of 7.9 and a *p*-value of 0.02. Given the nominal nature of the gender variable and the ordinal classification of financial toxicity, the Spearman correlation coefficient (*ρ*) was employed to measure the strength and direction of their correlation. Despite the weak correlation, a *ρ* value of -0.22 suggests a higher propensity for female patients to experience higher grades of financial toxicity (Table 2).

**Table 2:** A crosstab of relationships between sociodemographic variables and financial toxicity grade of the first round of data collection

Chi-Square Tests *N* = 99			
Variables	Financial toxicity grade		
	*χ*^*2*^ value	*P* value	Correlation coefficient
Sex (male – female)	7.9	0.02	-0.22
Dwelling status (not an IDP – an IDP)	8.4	0.02	0.23
Do you pay for cancer treatment (No – Yes)	6.1	0.11	0.16
Marital status (married – single – divorced – widow – no answer)	16.9	0.77	0.02
Education level (high education – secondary school – illiterate)	3.8	0.22	0.12
Employment status (employed – unemployed)	6.5	0.06	-0.18
Do you pay for accommodation (No – Yes)	3.6	0.06	0.18
Do you receive financial assistance from your family or relatives (No – Yes)	10.1	0.10	-0.17

The data show that IDPs and women are more likely to experience higher financial toxicity grades

**Fig. 1:**
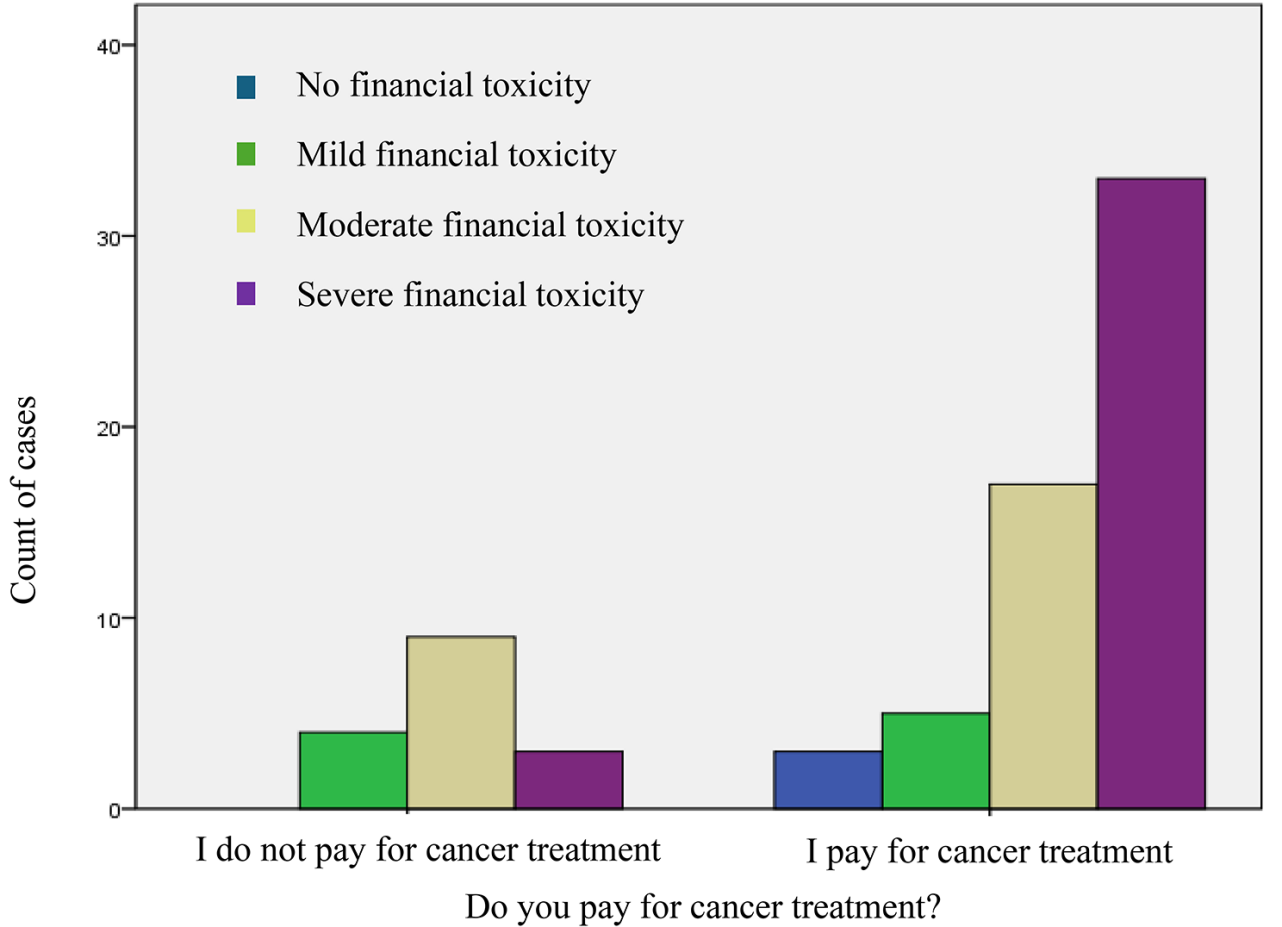
Financial toxicity grades for patients with an average monthly income of 87 USD or less (the first round of data collection)

Similar patterns were revealed when examining the correlation between dwelling status and financial toxicity grade (*χ*^*2*^ = 8.4, *P* = 0.02). The Spearman correlation value of 0.23 identified a weak but existent correlation between being an IDP and experiencing higher financial toxicity grades. The analysis did not demonstrate a significant relationship between financial toxicity grades and the payment for cancer treatments (*P* > 0.05). However, this relationship reached statistical significance when confounded by the factor of average monthly income (*P* < 0.05). We categorized the monthly income into two categories with a cutoff point of the average monthly income (87 USD). The results showed that patients who pay for cancer treatment and receive a lower monthly income are more likely to experience higher financial toxicity grades and, consequently, more subjective distress (Fig. 1).

The variables of marital status, education level, employment status, payment accommodation costs, and financial assistance from the family or relatives were not significantly related to financial toxicity grade.

## COST value: the second phase of data collection

The second phase of data collection encompassed 28 cancer patients who affirmed consistent receipt of humanitarian aid throughout the follow-up period. All the patients in the second phase completed the items in the COST-FACIT questionnaire. Out of those patients, 20 recorded COST values of moderate financial toxicity, 3 of mild financial toxicity, and 5 of no financial toxicity. The average COST value for the patients in the second phase was 13.82, which refers to a mild financial toxicity grade. The average age was 47.96 years, and the average monthly income was 80.54 USD. Of these cases, females constituted the majority (*n* = 21), with the predominant financial toxicity grade reported as moderate among them (*n* = 16) (Table 3). Additionally, most of the cases were not IDPs, married, illiterate, do not pay for cancer treatment, and receive financial assistance to cover life expenses and cancer therapy (Table 4).

**Table 3:** A crosstab of the sex of the patients and Financial toxicity grade (the second round of data collection)

		Financial toxicity grade			Total
		No Financial Toxicity	Mild Financial Toxicity	Moderate Financial Toxicity	
Sex of patient	Female	3	2	16	21
	Male	2	1	4	7
Total		5	3	20	28

**Table 4:** Demographics figures of the second round participants disaggregated by sex (the first round of data collection)

		Financial toxicity grade of the second round numerical. *N* = 28						Total
		No Financial Toxicity		Mild Financial Toxicity		Moderate Financial Toxicity		
		Sex of patient						
		Female	Male	Female	Male	Female	Male	
Dwelling status	an IDP	0	0	1	0	6	3	10
	Not an IDP	3	2	1	1	10	1	18
Marital status	No answer	0	0	0	0	1	0	1
	Married	3	2	2	1	10	4	22
	Single	0	0	0	0	3	0	3
	Widow	0	0	0	0	2	0	2
Level of education	Illiterate	2	0	0	0	13	1	16
	Secondary school	1	2	2	1	3	3	12
Employment	No	3	2	2	1	16	4	28
Do you pay for accommodation?	No	3	2	1	1	16	4	27
	Yes	0	0	1	0	0	0	1
Do you pay for cancer treatment?	No	0	0	0	0	1	0	1
	Yes	3	2	2	1	15	4	27
Do you receive financial assistance?	No answer	0	0	0	0	1	0	1
	No	0	2	1	1	5	2	11
	Yes	3	0	1	0	10	2	16

Because the sample size of the second phase was relatively small and assumptions underlying the chi-squared test could not be met, we applied the Fisher Exact test to investigate relationships between financial toxicity grades and sociodemographic variables [49] [50]. The Fisher Exact test did not show a statistically significant relationship between financial toxicity grade and any of the patients’ sociodemographic variables in the second phase except for sex (*P* < 0.05, CI: 95%).

### The impact of humanitarian assistance on financial toxicity

**Fig. 2:**
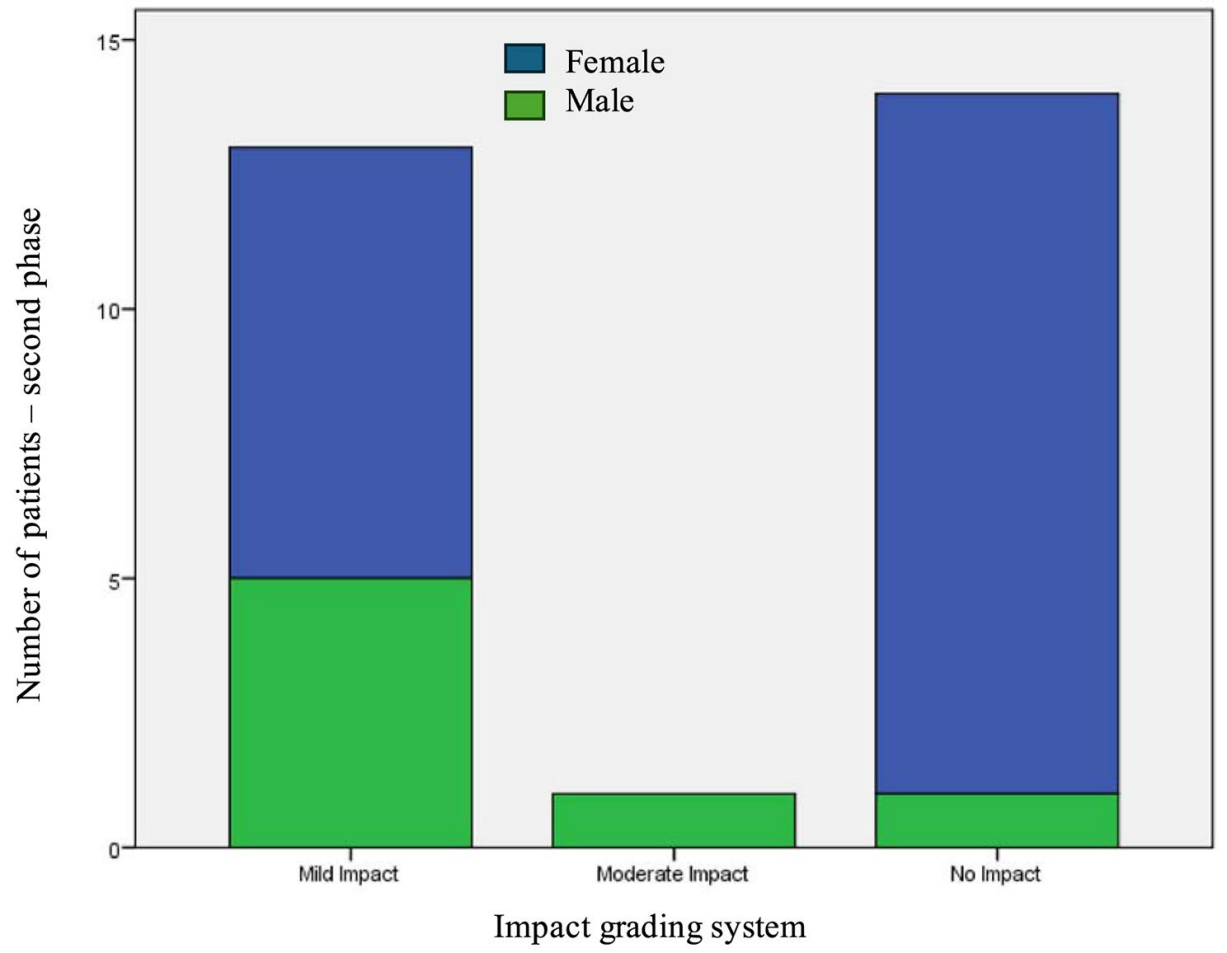
Impact grading system of humanitarian aid on financial toxicity categorized by sex. This chart illustrates the distribution of patients across different impact categories disaggregated by sex

The difference between the financial toxicity grades between the two phases was calculated for each patient, and the results were categorized based on the suggested Impact Grading System of Humanitarian Aid on Financial Toxicity. Data analysis revealed that regular humanitarian assistance had a negligible to mild impact on the financial toxicity levels (Table 5). Besides, it was found that financial toxicity among women was less impacted by humanitarian aid. Figure 2, a gender-segregated bar chart, shows distinct patterns in the impact of humanitarian aid on financial toxicity among male and female cancer patients in NWS. It highlights that while both genders predominantly experienced ‘No Impact’, a greater proportion of male patients reported ‘Mild Impact’ compared to their female counterparts.

**Table 5:** Impact grading system of humanitarian aid on financial toxicity. *N* = 28

		Frequency	Percent %
Impact grading system	No Impact	14	50.0
	Mild Impact	13	46.4
	Moderate Impact	1	3.6
	Total	28	100.0

Due to the non-normal distribution of financial toxicity variable in the second round using Shapiro-Wilk test for small samples below 50 observations (*p* < 0.05, 95% CI) [51], the Wilcoxon Signed-Rank test was employed to assess the median differences in COST value within the sample over the two phases and to determine the presence of any significant associations between the scores. The analysis revealed no significant association between the receipt of humanitarian aid and the level of financial toxicity across the two phases among cancer patients (*P* = 0.732), leading to the retention of the null hypothesis that humanitarian aid has no significant impact on the financial toxicity experienced by cancer patients in NWS.

## Discussion

The research addressed the intersection of financial toxicity and humanitarian aid, a niche yet crucial area within oncology in conflict-affected NWS. Despite the provision of humanitarian assistance, the study’s findings suggest that the impact on financial toxicity levels among cancer patients remains limited. This aligns with previous findings in the literature that financial toxicity is a multifaceted issue, often influenced by systemic healthcare inadequacies, socioeconomic instability, and individual patient variables [13] [52]. The lack of a substantial relationship between humanitarian aid and reduced financial toxicity underscores the complexity of financial toxicity as a construct and the potential inadequacy of current humanitarian models to address it within the cancer care paradigm. This statement is consistent with the findings presented by Hannah R Abrams et al. in their 2021 publication, which examines the economic challenges encountered by cancer patients. The article discusses methods for alleviating financial toxicity, enhancing access to high-value medical care, and addressing healthcare inequalities [53].

While certain healthcare services may be accessible in NWS by cancer patients, the limited availability of these services requires cancer patients to seek medical care in Türkiye. This requirement presents a growing challenge, further burdening patients with additional expenses related to travel and accommodation [30]. These challenges are exacerbated, especially after the 2023 earthquake, by the high poverty rates, poor socioeconomic conditions, and the lack of sustainable health economic measures such as comprehensive medical insurance [54] [55]. In settings affected by conflict and economic instability, the interplay between healthcare limitations, economic collapse, and logistical challenges intensifies the financial toxicity experienced by patients. For instance, Borah et al. (2022) emphasize the exacerbated financial and psychological hardships in conflict-affected regions, where the lack of resources and increased economic strain profoundly impact individuals, families, and governments [56]. While the reasons for financial toxicity in the context of NWS are similar to other contexts, the current instability, collapse and fragmentation of the healthcare system, and the low efficiency of humanitarian assistance in addressing this issue further exacerbate the financial distress experienced by cancer patients [57]. Abdul-Khalek et al. (2020) highlight the high costs of cancer treatment for Syrian refugees in Lebanon, Jordan, and Türkiye, emphasizing the burden increasingly placed on patients, which results in catastrophic consequences for health and quality of life [58]. In general, the analysis of financial toxicity in oncology care across various contexts highlights a similarly complex situation, further intensified in conflict-affected regions due to additional barriers to care. The literature emphasizes the necessity for a comprehensive approach to funding and support, focusing on policy reforms, patient education, and systemic changes in humanitarian healthcare delivery [59] [60]. These measures are crucial in mitigating the financial burdens experienced by oncology patients. However, The reliance on a single-region sample may limit the generalizability of our findings to other conflict-affected areas with different socio-political dynamics and healthcare infrastructure.

The research findings suggest a complex interaction between demographic factors and financial toxicity among cancer patients in NWS, a region characterized by ongoing conflict and humanitarian crises [57]. The significant relationship between gender and financial toxicity, with a higher likelihood for female patients to experience more severe financial distress, is notable. This gender disparity may reflect broader societal patterns of economic disadvantage among women in LMICs and is compounded by the conflict in Syria. The study’s revelation of the dwelling status as a significant factor adds another layer to the financial toxicity issue. IDPs, often residing in camps or informal dwellings, are at a higher risk of severe financial toxicity, suggesting that instability and lack of housing contribute to economic strain. These findings are critical as they point to broader social inequalities exacerbated in crisis conditions, which humanitarian aid efforts must address more effectively.

The study’s core inquiry into the impact of humanitarian aid on financial toxicity reveals several findings that should be addressed by the humanitarian aid system in NWS. While humanitarian aid is traditionally geared towards immediate life-saving interventions and basic needs provision, its role in mitigating the mid-term financial effects of cancer treatment is less evident. This research indicates that humanitarian assistance, as currently structured, does not significantly alleviate the financial toxicity experienced by cancer patients in NWS. Despite this, the role of humanitarian aid cannot be discounted entirely; it may provide a foundational support system that, if restructured, could potentially address broader financial needs.

In general, the data analysis did not show a statistically significant relationship between receiving humanitarian assistance and improvements in financial toxicity grades. This suggests that while aid may relieve some immediate financial pressures, it does not translate into a substantial decrease in the overall economic burden that cancer treatment poses. It is, therefore, crucial to explore why humanitarian aid fails to address this aspect of financial distress and how aid mechanisms can be refined.

Our study is distinguished by its innovative investigation into the impact of humanitarian aid on financial toxicity among cancer patients in NWS, a topic that has received limited attention in existing literature. This research sheds light on the intricate relationship between conflict, healthcare accessibility, and economic hardship, offering invaluable insights into the complex challenges faced by oncology patients in regions affected by conflict. The use of the COST-FACIT tool, specifically adapted to the Arabic language and cultural context, and recruiting a high proportion of female patients enhances the credibility of our findings by providing a reliable measure of financial distress among a highly vulnerable population. Additionally, the study’s quasi-experimental design enables a focused analysis of the pre and post-effects of humanitarian interventions on financial toxicity, thereby contributing significant empirical evidence to the field.

## Conclusion

Our study presents a novel and essential analysis of financial hardship in conflict-affected settings, drawing attention to the issue of “data poverty” and underscoring the importance of comprehensive data for informing humanitarian and healthcare policy-making. While the contribution of aid in alleviating immediate distress is recognized, its efficacy in addressing the enduring financial strains of cancer treatment remains uncertain.

Key findings indicate a critical need to rethink humanitarian aid models in NWS, with a focus on chronic diseases like cancer, particularly affecting female patients. Proposed strategies include integrating economic strengthening activities, cash assistance, vocational training, and educational initiatives to alleviate the indirect costs associated with cancer care. The study also underscores the necessity of multi-sectoral collaboration, suggesting that healthcare delivery and economic support must be closely aligned to effectively address the issues of health and financial stability in conflict zones. Furthermore, it advocates for expanding humanitarian programs to encompass financial counseling, subsidies for treatment costs, and microfinance opportunities to address the economic impact of healthcare disruptions caused by conflict.

Our conclusion that humanitarian aid has no significant impact on financial toxicity is a strong claim that may not be fully substantiated by the data presented. Therefore, it is imperative to undertake further research to explore the underlying causes of this lack of impact more comprehensively and develop a systematic pathway from research findings to policy recommendations. The findings of this study suggest that while humanitarian aid is beneficial for patients, the availability of sustainable medical interventions and therefore the need for a strong health system to address local needs is essential.

It is worth noting that applying the COST tool in the context of a humanitarian crisis may not capture all dimensions of financial toxicity experienced by patients in such settings. Therefore, the external validity of this tool in emergency contexts must be established. As the first study to investigate this topic in Syria and globally within a conflict context, it lays the groundwork for future exploration and sets a precedent for empirical assessment of humanitarian aid’s impact on health economics. The study’s findings also advocate for the need to rethink humanitarian assistance delivery, aiming for a model that integrates financial protection strategies for vulnerable populations facing high-cost chronic diseases amidst crises.

### Electronic supplementary material

Below is the link to the electronic supplementary material.

Supplementary Material 1

Supplementary Material 2

## Data Availability

The datasets generated and/or analyzed during the current study are available on the Mendeley Data website: https://data.mendeley.com/datasets/dsktsd99g3/1.

## Abbreviations

LMIC: Low and Middle-Income Countries
COST: Comprehensive Score for Financial Toxicity
NWS: Northwest Syria
FACIT: Functional Assessment of Chronic Illness Therapy
IDP: Internally Displaced Person
