# Supplementary material for: The impact of humanitarian aid on financial toxicity among cancer patients in Northwest Syria
Source: BMC Health Serv Res. 2024 May 18;24:641. doi: 10.1186/s12913-024-11077-x (PMC11102167; doi:10.1186/s12913-024-11077-x)
Supplement: Supplementary file 1 — Supplementary Material 1 [file 12913_2024_11077_MOESM1_ESM.pdf]

Annex 1

Questionnaire

The Impact of Humanitarian Aid on Financial Toxicity Among Cancer Patients in Northwest Syria – (Kuzeybatı Suriye'deki Kanser Hastalarında İnsani Yardımın Finansal Toksisite Üzerindeki Etkisi) Anket Soruları

Before the questionnaire, please read the explanation below.

Dear participant,

The questions you will answer later are about the application part of the scientific research called "The Impact of Humanitarian Aid on Financial Toxicity Among Cancer Patients in Northwest Syria". The research is completely academic, and the information obtained from the study will be used for scientific purposes, and the answers will be kept strictly confidential.

We thank you in advance for your valuable contribution to the study.

- **The hospital or medical center name:**
- **The hospital or medical center address:**
- **The names of the data collector team:**

- **First Stage:**

1- Age (years): (If less than 18 years, please end the survey- exclusion criteria)
2- Did you have a confirmed diagnosis of any malignant tumor of any stage at the existing Oncology centers in NWS (Yes-no) (If no, please end the survey- exclusion criteria)
3- Did you receive humanitarian non-medical assistance in the past 2 months? (Yes – no) (If yes, please call off the survey- exclusion criteria)
4- Since when are you receiving medical therapy for your case (in months)? (If less than two months, please call off the survey – exclusion criteria)
5- Name: (to be converted to a unique ID)
6- Sex (male-female)
7- Race (Arabic – Kurdish – Turkman – Other).
8- Place of residence (governorate – community).
9- Dwelling status (Internally Displaced Person IDP, or not an IDP).
10- If IDP, type of residency (camp and informal residency settings – dignified housing).
11- Marital status (married, single, divorced, widow).
12- Level of education (university and higher education, Intermediate Institute, secondary school education or lower, illiterate).
13- Employed (yes – no)
    a. If yes, what is your current profession?
14- Do you pay for cancer treatment? (yes – no).

15- If yes, do your family or relatives financially support you to cover the treatment or life expenses? (yes – no)

16- Average monthly income per household in USD (*if the income was expressed in other currencies, please convert it to USD using the corresponding exchange rate according to the researcher's instructions*). (*If the patient cannot identify the household's average monthly income, you can ask his/her adult guardian*).

Now proceed to the COST questionnaire.

- **Second Stage:**

17- Did you receive humanitarian non-medical assistance in the past 7 days? (yes – no)

18- If yes, what was the type of assistance? Please select all valid choices (cash vouchers or cash distribution – food distribution – hygiene kits – non-food items)

Now proceed to the COST questionnaire.
